# Using mass spectrometry imaging to visualize age-related subcellular disruption

**DOI:** 10.3389/fmolb.2023.906606

**Published:** 2023-03-08

**Authors:** Kelly A. Hogan, Julianna D. Zeidler, Heather K. Beasley, Abrar I. Alsaadi, Abdulkareem A. Alshaheeb, Yi-Chin Chang, Hua Tian, Antentor O. Hinton, Melanie R. McReynolds

**Affiliations:** ^1^ Department of Biochemistry and Molecular Biology, Pennsylvania State University, University Park, PA, United States; ^2^ Signal Transduction and Molecular Nutrition Laboratory, Kogod Aging Center, Department of Anesthesiology and Perioperative Medicine, Mayo Clinic College of Medicine, Rochester, MN, United States; ^3^ Huck Institutes of the Life Sciences, Pennsylvania State University, University Park, PA, United States; ^4^ Instituto de Bioquímica Médica Leopoldo de Meis, Universidade Federal do Rio de Janeiro, Rio de Janeiro, Brazil; ^5^ Department of Molecular Physiology and Biophysics, Vanderbilt University, Nashville, TN, United States; ^6^ Department of Chemistry, Pennsylvania State University, University Park, PA, United States

**Keywords:** mass spec imaging, MERCs, Golgi complex, mitocchondrial dysfunction, aging, metabolic flux, spatial omics

## Abstract

Metabolic homeostasis balances the production and consumption of energetic molecules to maintain active, healthy cells. Cellular stress, which disrupts metabolism and leads to the loss of cellular homeostasis, is important in age-related diseases. We focus here on the role of organelle dysfunction in age-related diseases, including the roles of energy deficiencies, mitochondrial dysfunction, endoplasmic reticulum (ER) stress, changes in metabolic flux in aging (e.g., Ca^2+^ and nicotinamide adenine dinucleotide), and alterations in the endoplasmic reticulum-mitochondria contact sites that regulate the trafficking of metabolites. Tools for single-cell resolution of metabolite pools and metabolic flux in animal models of aging and age-related diseases are urgently needed. High-resolution mass spectrometry imaging (MSI) provides a revolutionary approach for capturing the metabolic states of individual cells and cellular interactions without the dissociation of tissues. mass spectrometry imaging can be a powerful tool to elucidate the role of stress-induced cellular dysfunction in aging.

## Introduction

Aging is the greatest risk factor for the most prevalent diseases in industrialized nations. The coordinated action of energetic molecules ensures metabolic homeostasis, normal cellular function, and increased healthspan. The cellular hallmarks of aging are epigenetic alterations, loss of proteostasis, mitochondrial dysfunction, cellular senescence, and exhaustion of stem cells (Ziegler et al., 2021). However, dysfunctional organelles are increasingly linked to diseases of aging such as chronic metabolic disorders, neurodegenerative diseases, and shortened lifespan (Ziegler et al., 2021). Among organelles ripe for interrogation in the aging process are the mitochondria, the endoplasmic reticulum (ER), and the sites of inter-organelle communication called mitochondria-ER contact sites (MERCs). MERCs are enriched with proteins essential for mitochondrial Ca^2+^ flux, lipid transfer, autophagy, mitochondrial division, apoptosis, and morphology (Giacomello and Pellegrini, 2016; Csordás et al., 2018), and these contact sites may be important in understanding how metabolic fluxes are altered during the aging process. However, studies to define the role of MERCs in metabolic fluxes using single cell techniques such as mass spectrometry imaging (MSI) are lacking.

In addition to well characterized calcium fluxes between the ER and mitochondria, there are other metabolic exchanges including those associated with nicotinamide adenine dinucleotide (NAD^+^) and precursors to NAD^+^ synthesis that are strongly associated with diseases of aging (McReynolds et al., 2020; McReynolds et al., 2021; Zeidler et al., 2022). NAD^+^ is a cofactor for redox and non-redox enzyme reactions that maintain metabolic pathways, DNA repair, chromatin remodeling, immune cell function, and cellular senescence (Hogan et al., 2019; Covarrubias et al., 2021). A decline in cellular NAD^+^ levels is linked to several diseases of aging in mice including neurodegeneration and cognitive decline, cancer, metabolic disease, sarcopenia, frailty, hearing loss, stroke, cataracts, and kidney and heart disease (Hogan et al., 2019; Covarrubias et al., 2021). Senescent cells secrete pro-inflammatory factors characteristic of the senescence-associated secretory phenotype (SASP), resulting in the accumulation of CD38^+^ inflammatory cells (Chini et al., 2020; Covarrubias et al., 2020). As an NADase and nicotinamide mononucleotidase (NMNase), CD38 decreases cellular NAD^+^ and extracellular NMN, respectively, and contributes to low-grade inflammation called inflammaging (Franceschi et al., 2018). The role of NAD^+^ metabolism in the regulation of SASP is not well understood, but both *de novo* and salvage NAD^+^ synthesis pathways are implicated in senescence. For example, the rate-limiting NAD^+^ synthesis salvage pathway enzyme nicotinamide phosphoribosyltransferase (NAMPT) promotes SASP through nuclear factor kappa B (NF-κB) in IMR90 fibroblasts (Nacarelli et al., 2019). Additionally, disruption of the *de novo* NAD^+^ synthesis pathway may lead to a decline in NAD^+^ in macrophages and innate immune cell dysfunction observed in aging and age-related diseases (Minhas et al., 2019). Single-cell technologies that combine imaging and metabolomics to interrogate inter-organellar trafficking of molecules like NAD^+^ are potentially powerful tools for understanding the aging process at the cellular and subcellular levels. Defining the metabolic landscape and the variability within cells and across cell types represents a new frontier in metabolomics and an opportunity to re-envision our understanding of cellular biology.

The emerging field of single-cell “spatial omics” resolves genomics, proteomics, and transcriptomics associated with intracellular structures in tissue sections without dissociating tissues or disturbing cellular interactions (Blum et al., 2018; Gilmore et al., 2019; Peng et al., 2020; Bai et al., 2021; Stein et al., 2021; Taylor et al., 2021). However, resolving the spatial omics for metabolites important in the pathophysiology of diseases has been more challenging. For example, although lipids play a central role in signaling, regulation, inflammation, and cancer, preserving the spatiotemporal gradient of these dynamic and transient small molecules is difficult. Here, we propose that spatial omics can provide a better understanding of organellar metabolism to identify the mechanisms of cellular decline during aging. Although typical age-related processes such as autophagy and apoptosis may be regulated through age-associated MERC modifications, changes in the composition or number of MERCs may also be pro-senescent (Ziegler et al., 2021). This review focuses primarily on the structure, function, and role of mitochondria and the ER in age-related diseases, with special emphasis on MERCs as modifiers or targets of cellular aging (Janikiewicz et al., 2018; Csordás et al., 2018; Moltedo et al., 2019; Ziegler et al., 2021).

Single-cell technologies that combine imaging and metabolomics to interrogate inter-organellar trafficking of molecules, like NAD^+^, are potentially powerful tools for understanding the aging process at the cellular and subcellular levels. Defining the metabolic landscape and the variability within cells and across cell types represents a new frontier in metabolomics and an opportunity to re-envision our understanding of cellular biology. However, few studies have defined the role of MERCs in metabolic fluxes using single-cell techniques, such as mass spectrometry imaging (MSI). We also describe the advantages and limitations of MSI, particularly as a tool to interrogate age-related dysfunction at the single-cell level.

## Spatial metabolomics/lipidomics as a tool for interrogating diseases of aging

Cell-to-cell variation is intrinsic to cell populations, organs, and tumors and includes differences in epigenetic, transcriptional, translational, and metabolic regulation; therefore, understanding these differences is important for defining age-related regulatory changes across cell and tissue types. Identifying spatially resolved age-related metabolic alterations will be key to elucidating the impact of these changes on the aging trajectory and developing effective metabolite-targeted treatments for age-related diseases. Changes in metabolite levels triggered by fluctuations in the cellular environment or altered intracellular signaling are reliable markers of cellular status. Single-cell transcriptomics provides powerful insights into differences observed in normal and pathological contexts. Likewise, studies of the metabolome in single cells are also likely to provide a comprehensive profile of individual cellular changes associated with age. Spatial and temporal snapshots of nutrient gradients, and fluxes in particular, may become the basis of discovery of new interventions for aging-related diseases.

**FIGURE 1:**
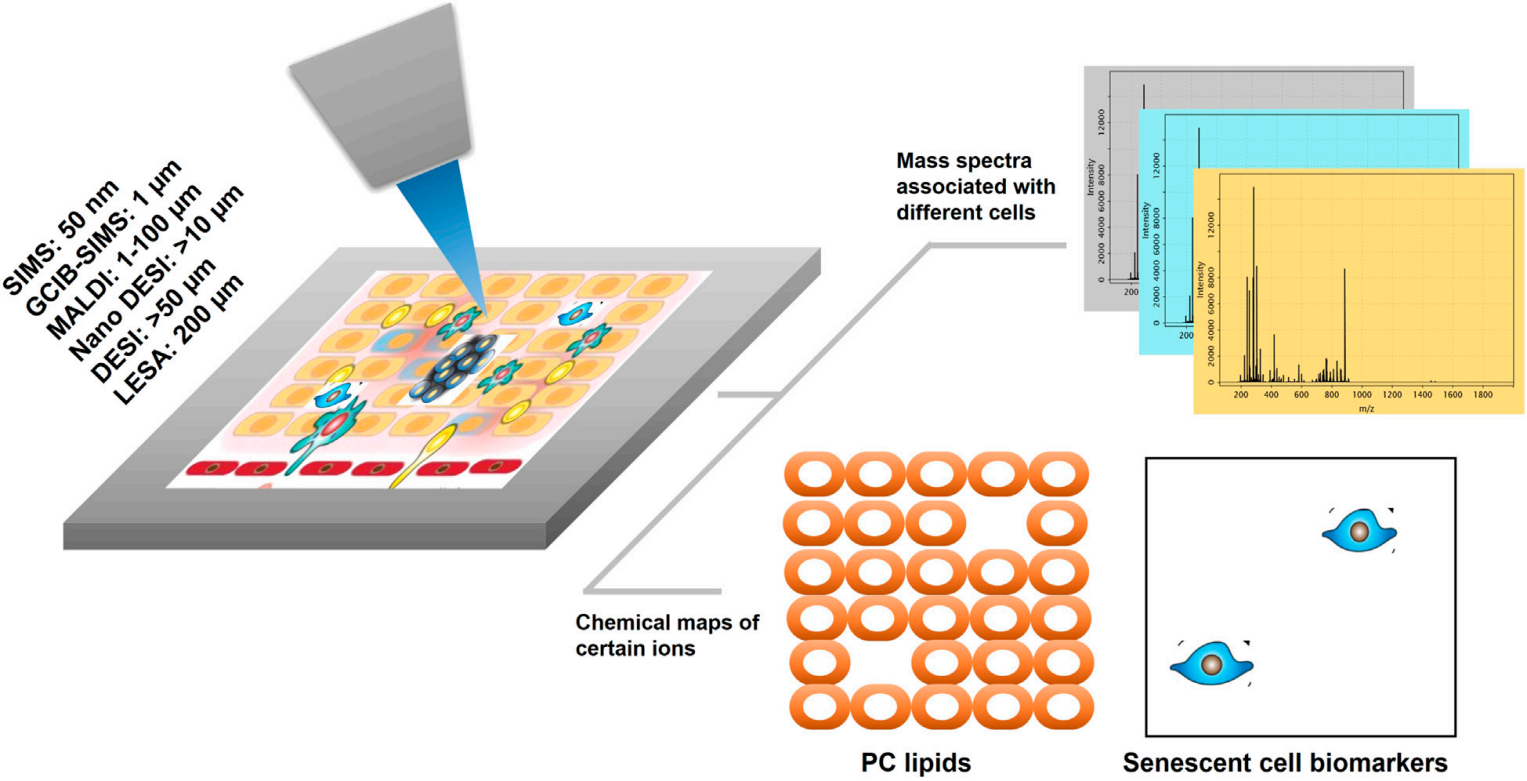
A simplified workflow of MSI for spatial omics. The focused ion desorption source scans the tissue surface (or cells in culture) pixel by pixel. Both mass spectra and x and y coordinates are generated from each pixel. With the single-cell resolution of the ion desorption source, omics information is obtained from individual cells in the tissue or cell culture without tissue dissociation. Biomolecules or biomarkers are localized to cellular structures or different cell populations. Technical approaches for MSI workflows include: SIMS, secondary ion mass spectrometry; GCIB, gas cluster ion beam; MALDI, matrix-assisted laser desorption ionization; DESI, desorption electrospray ionization; LESA, liquid extraction surface analysis.

**FIGURE 2:**
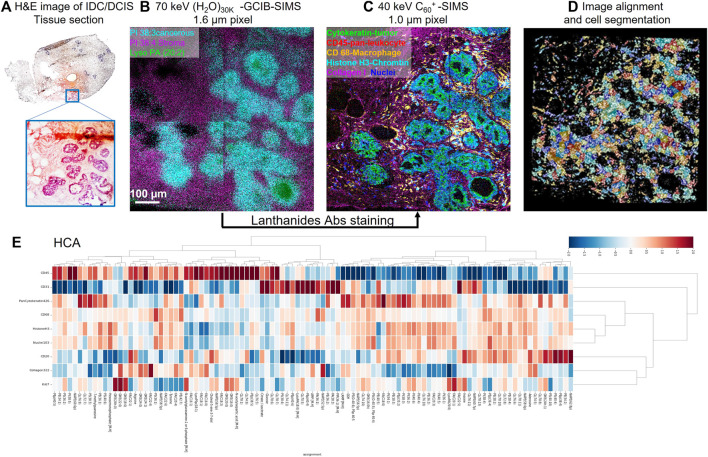
Schematic of the workflow for cell-type-specific profiling of multi-omics on IDC/DCIS tissue sections at the single-cell level with select overlays and single-ion images using (H_2_O)_n_-GCIB-SIMS and C_60_-SIMS. **(A)** H&E staining image of a semi-serial section of invasive ductal carcinoma/ductal carcinoma *in situ* (IDC/DCIS) tissue. The enlarged image from the region of interest highlighted in blue shows the tumor region in purple and the stromal region in pink. **(B)** (H_2_O)_n_-GCIB-SIMS imaging of a fresh-frozen tissue section. The cryogenic analysis at 100 K was performed on frozen-hydrated tissue sections for molecular imaging (e.g., lipids and metabolites) at a beam spot size of 1.6 µm. **(C)** C_60_-SIMS imaging of the same tissue section stained with a lanthanide-tagged cocktail of antibodies at a beam spot size of 1.0 µm. **(D,E)**, **(D)** the image alignment and cell segmentation were used to integrate the omics **(E)** metabolites, lipids, and proteins into different cell types at single-cell resolution.

MSI is a tool that has the potential to visualize pro-senescent MERCs because the technology has the capacity to simultaneously detect proteins, lipids, and metabolites. To date, there are several technical approaches and workflows for MSI including gas cluster ion beam secondary ion mass spectrometry (GCIB-SIMS), matrix-assisted laser desorption ionization (MALDI), desorption electrospray ionization (DESI), and liquid extraction surface analysis (LESA) (Figure 1). GCIB-SIMS is a particularly promising approach to spatially mapping the age-related metabolites in single cell or subcellular organelle level. With an innovative new desorption source, high-energy CO_2_ or H_2_O cluster ion beam, GCIB-SIMS resolves biomolecules at the lateral resolution of 1 µm without comprising signal level of low concentration biomolecules (e.g., transient metabolites). Among several advantages of the GCIB source is enhanced ionization up to ∼200 fold to facilitate detection of biomolecules; reduced ionization suppression to allow the imaging of diverse biomolecules; and extension of mass detection ranges for spatial multiomics in a single sample (Tian et al., 2014; Wucher et al., 2014; Tian et al., 2016a; Tian et al., 2016b; Tian et al., 2016c; Tian et al., 2016d; Tian et al., 2017a; Tian et al., 2019a; Tian et al., 2019b; Sheraz et al., 2019). Coupled with a unique buncher-ToF SIMS instrument (J105 SIMS) (Fletcher et al., 2008; Tian et al., 2016d), GCIB-SIMS achieves multi-omics imaging at cellular/subcelluar resolution in biological samples (Tian et al., 2019a; Tian et al., 2021a). Additionally, as one of very few MSIs capable of cryogenic analysis, GCIB-SIMS captures images of frozen-hydrated single cells and preserves the chemical microenvironments at near-nature state with minimal disruption of the tissue and cellular structures, especially for the dynamic metabolic gradients (Tian et al., 2014; Tian et al., 2016b; Tian et al., 2019a; Tian et al., 2021a). Additionally, GCIB-SIMS produces molecular species, (M + H)^+^ or (M-H)^−^ for biomolecules, as well as different forms of fragments. A caveat, however, are incompatibilities in sample preparation that prevent the GCIB-SIMS cryogenic workflow from being adapted for other MSI tools, thus making them unsuitable for exploration of spatial metabolomics (Robinson and Castner, 2013; Zhang et al., 2018). As the first multiplexing pipeline to integrate multiomics in the same cells, the GCIB-SIMS multimodal imaging platform simultaneously detects metabolites and lipids with multiple protein expression markers in frozen-hydrated breast cancer tissue and mouse liver tissue at the single-cell level (Tian et al., 2021b). As shown in Figure 2 (Tian et al., 2021b), untargeted metabolites/lipids and multiple targeted proteins are imaged at 1 µm spatial resolution in human breast cancer tissue, facilitating cell segmentation and registration of multi-omics in individual cells by computational processing and discriminant analysis. The workflow permits visualization of multiple molecular coordination (>150 key species) in different types of tumor microenvironment cells including actively proliferating tumor cells and infiltrating immune cells. For example, desaturated fatty acids, phosphatidylethanolamine plasmalogen and glutathione are observed in pan cytokeratin-expressing cells (i.e., epithelial cancer cells), suggesting that cancer cells adopt antioxidant mechanism to prevent lipids oxidation-induced ferroptopic cell death under conditions of stress.

To this end, the GCIB-SIMS workflow integrates spatial omics at the single-cell level, providing a new approach for visualizing individual cells, cellular interactions, and chemical microenvironments in aging tissues for deeper insights into the mechanisms of age-related diseases. Spatially resolved approaches to elucidating metabolic activity will be particularly important for highly age-sensitive epithelial, mesenchymal, immune, senescent, and cancer cells, as well as for cells from relatively short-lived gestational tissues at the maternal-fetal interface. As demographics continue to shift toward an aging population and diseases of aging grow more prevalent, tools to interrogate compromised tissue microenvironments and disrupted cell-to-cell communication are critical for identifying druggable targets in age-related diseases. Metabolic shift in different types of cells within the tissue is vital to understanding aging-prone cell types. With high sensitivity for various biomolecules, GCIB offers a soft ionization for imaging of pathways involving molecules like NAD^+^ with the potential to ionize NADP^+^ from NADPH as distinct molecular ions. MSI, especially high-resolution GCIB-SIMS, could transform current understanding of aging tissues by: 1) mapping the variation of metabolic flux resulting from NAD^+^ synthesis and degradation in individual cells in tissues vulnerable to NAD^+^ decline; 2) characterizing cellular heterogeneity within tissues to understand mechanisms of inflammaging and the impacts of inflammation on neighboring cell types; 3) identifying metabolic imbalances and reprogramming in age-related diseases; and 4) resolving metabolic gradients in subcellular organelles, including NAD(H) and NADP(H) trafficking across MERCs.

## Organelles targeted by age-associated energy deficiencies

### The aging mitochondria

Mitochondria govern cellular homeostasis by coordinating the energetic needs of the cell. Mitochondrial dysfunction, a hallmark of aging, underlies several neurological disorders and disorders of metabolism such as obesity, aging, hypertension, and type 2 diabetes (Ihab Hajjar and Kotchen, 2006; Esther Phielix and Roden., 2011; Rieusset, 2018). The pathophysiology resulting from deterioration of mitochondrial function likely contributes to the age-dependent decrease in organ function driven by loss of energy production, lipid biogenesis, and activation of cell death programs (Ian Lanza and Nair, 2010; Miriam Valera-Alberni and Canto, 2018; Seungyoon Yu and Pekkurnaz, 2018). Signals necessary for maintaining cellular homeostasis are exchanged continuously between mitochondria and ER through organelle-linking contact sights known as mitochondria-ER contact sites (Csordás et al., 2018) or MERCs. Namely, the transfer of Ca^2+^ from ER to mitochondria is vital for maintaining mitochondrial energy metabolism. MERC distance or thickness is integral to normal Ca^2+^ signaling and transport (Giacomello and Pellegrini, 2016; Csordás et al., 2018). Contact sites outside of the 12–24 nm range appear to alter Ca^2+^ uptake and lipid dynamics, in this context (Giacomello and Pellegrini, 2016), by creating steric hindrance that disrupts Ca^2+^ transport machinery within MERCs (Giacomello and Pellegrini, 2016). Mitochondrial Ca^2+^ is required for the activation of oxidative phosphorylation (Roberto Bravo et al., 2011; Brian Glancy and Balaban, 2012; Rieusset, 2018) and dysregulation of mitochondrial Ca^2+^ homeostasis may lead to higher amounts of radical oxygen species (ROS) (Artyom Baev et al., 2022).

Mitochondrial ROS contributes to age-related loss of tissue homeostasis (Shigenaga et al., 1994; Carlos López-Otín et al., 2013) and changes to MERCs are likely to target the “redox triangle” which includes the mitochondria, ER, and peroxisomes. Mitochondrial ROS arises from the leakage of electrons from redox centers of respiratory complexes and associated enzymes (Hoi-Shan Wong et al., 2017). There are at least eleven sites of O_2_^•–^ and H_2_O_2_ generation in mitochondria. The rates of ROS generation and the relative contribution of each site depends on substrate availability and cellular metabolic context (Hoi-Shan Wong et al., 2017). What specifically triggers increased mitochondrial ROS during aging is still under debate, but roles for the initiation of senescence by mitochondrial ROS have been established (Passos et al., 2010; Clara Correia-Melo et al., 2016). For example, excessive production of mitochondrial ROS can drive telomeric-induced senescence (Passos et al., 2007) and oncogene-induced senescence (Olga Moiseeva et al., 2009). ROS also contribute to cellular senescence by promoting DNA damage (Passos et al., 2010), which in turn activates stress-response pathways such as JNK1/2, that ultimately leads to SASP formation (Maria Grazia Vizioli et al., 2020), NLRP3 inflammasome activation (Rongbin Zhou et al., 2011), in addition to changes in mitochondrial structure, function, and dynamics (Hélène Martini and Passos, 2022). Thus, mitochondria play essential roles in establishing and maintaining the senescence phenotype with production of mitochondrial ROS as a key component.

In addition to causing DNA damage, ROS also promote lipid peroxidation, leading to decreased mitochondrial membrane fluidity and dysregulated mitochondria function (Chen and Yu, 1994; Shigenaga et al., 1994). Increased ROS can also lead to protein oxidation. A quantitative “oxi-proteome” analysis has identified oxidized proteins enriched in senescent fibroblasts by measuring 4-hydroxy-2-nonenal (HNE)-, glycoxidation (AGE)-, and carbonylation-modified proteins (Emad Ahmed et al., 2010). Consistent with increased mitochondrial ROS in senescence, half of these oxidized proteins were mitochondrial with most involved in energy metabolism and protein quality control (Emad Ahmed et al., 2010). Not surprisingly, proteins from the cytoplasm, nucleus, and ER are also vulnerable to oxidation (Emad Ahmed et al., 2010). By mapping oxidized biomolecules such as lipids and proteins, MSI could be used to determine the extent to which oxidative damage derived from mitochondrial ROS spreads throughout cells and alters metabolism in surrounding organelles or neighboring cells. This approach could help answer foundational questions concerning the role of mitochondrial ROS in aging and identify both sources and consequences of ROS in aged tissues.

The tricarboxylic acid (TCA) cycle intermediates are another link between mitochondrial metabolism and the senescence phenotype. Some TCA cycle metabolites play pleiotropic roles outside mitochondria to influence epigenetic modification, immune regulation, and cell signaling. For instance, cytosolic citrate is required to produce acetyl-CoA for histone acetylation (Wellen et al., 2009) and can act as an inflammatory signal (Vittoria Infantino et al., 2011). α-Ketoglutarate is a co-factor for 2-oxoglutarate/Fe^2+^-dependent dioxygenases (OGDD). These enzymes catalyze the hydroxylation of various substrates such as nucleic acids, lipids, and proteins, including histone demethylases in the nucleus (Inmaculada Martínez-Reyes and Chandel, 2020). Also, succinate accumulation can inhibit the activity of OGDDs and HIF-prolyl hydroxylases (HPH) and promote DNA hypermethylation and pseudohypoxia, respectively (Elaine MacKenzie et al., 2007; Eric Letouzé et al., 2013). Senescent cells display increased levels of TCA cycle metabolites such as citrate, α-ketoglutarate, and malate likely as a result of impaired mitochondrial function (Dörr et al., 2013; Joanna Kaplon et al., 2013; Emma et al., 2015). *In vivo*, α-ketoglutarate supplementation improves the lifespan and healthspan of old mice, reduces systemic inflammation, increases bone mass, and attenuates age-related bone loss (Inmaculada Martínez-Reyes and Chandel, 2020; Yuan Wang et al., 2020). In humans, citrate levels increase in the circulation during chronological aging (Cristina Menni et al., 2013; Kirsi Auro et al., 2014) and the potential implications of citrate in age-related diseases are reviewed elsewhere (Mycielska et al., 2022). Thus, the TCA cycle metabolome seems to be disrupted in cellular senescence and aging, but the consequences at the molecular level remain mostly elusive. MSI may resolve trace changes in the subcellular localization of TCA cycle metabolites in tissues during aging and provide clues to the functions those metabolites serve in age-related tissue dysfunction.

What causes age-related defects in mitochondria is still under intense investigation. Although multifactorial and context-dependent, evidence points to disrupted NAD^+^ metabolism as a player in mitochondrial decay. The primary cause of NAD^+^ decline in aging is increased NAD^+^ degradation without compensatory upregulation of NAD^+^ synthesis pathways (Camacho-Pereira et al., 2016; McReynolds et al., 2021). Upregulation of expression and increased activity of the NADase CD38 in old mice causes NAD^+^ levels to decline and affects the activity of Sirt3 (Camacho-Pereira et al., 2016). This mitochondria-localized sirtuin regulates mitochondrial metabolism and oxidative homeostasis. Thus, the CD38-NAD-Sirt3 axis leads to age-related mitochondrial dysfunction (Camacho-Pereira et al., 2016). In addition, NAD^+^ precursor supplementation improves mitochondrial function in aged mice and *C. elegans* (Gomes et al., 2013; Mouchiroud et al., 2013; Zhang et al., 2016), and overexpression of Nmnat3, an enzyme of the NAD^+^ salvage pathway, restores TCA cycle capacity and suppresses mitochondrial ROS generation in skeletal muscle of aged mice (Maryam Gulshan et al., 2018). However, only recently, SLC25A51 was identified as the mitochondrial NAD^+^ transporter (Girardi et al., 2020; Kory et al., 2020; Luongo et al., 2020), but little is known about the kinetics and regulation of NAD^+^ and NAD^+^ precursor influx into mitochondria in a physiological context and during aging. Flux analysis reveals incomplete equilibration of NAD^+^ between cytosol and mitochondria (McReynolds et al., 2021), suggesting that this organelle can be particularly sensitive to disturbances in cellular NAD^+^ levels. To uncover the consequences of disruptions in this dynamic during aging, MSI will likely be a powerful approach to characterizing NAD^+^ metabolome fluxes in and out of mitochondria.

### Endoplasmic reticulum (ER)

Communication between the ER and mitochondria is tightly regulated by mitochondria associated membranes (Zhang et al., 2021). ER homeostasis drives biosynthetic pathways including lipogenesis and gluconeogenesis (Gansemer and Rutkowski, 2022). The ER is composed of a set of membranes or cisternae that are held together by the cytoskeleton. The lumen of the ER stores phospholipids which are trafficked to various organelles. The volume of the rough or smooth ER lumens are dependent on the metabolic activity of the cell. ER functions include folding and transport of proteins to the Golgi apparatus, lipid biosynthesis, and Ca^2+^ storage *via* Ca^2+^-dependent chaperones. In the aging cell, ER chaperones are especially sensitive to oxidative damage and vulnerable to reduced Ca^2+^ buffering capacity, resulting in the deposition of insoluble fibrils or plaques as a consequence of oxidative stress (Balchin et al., 2016). Deposits that collect in the liver, brain, and spleen are pathognomonic for diseases associated with aging including Alzheimer’s disease, Parkinson’s disease, and Type 2 diabetes (Tian et al., 2019b; Sheraz et al., 2019).

In addition to oxidative stress, the ER responds to various metabolic stressors by triggering the unfolded protein response (UPR). Among UPR-inducing stressors, glucose deprivation leads to ER stress which interferes with disulfide bond catalysis and N-linked protein glycosylation with implications for caspase activation (Xu et al., 2005). Additionally, aberrant Ca^2+^ signaling in the ER causes improper protein folding, further contributing to the UPR. The UPR is an adaptive response that permits the cell to adjust to a challenging environment and maintain normal ER function and homeostasis. Adaptation also involves transcriptional modulation to enhance protein folding and removal of misfolded proteins in the ER. The UPR is primarily a cell-signaling system to maintain normal cellular function by restoring protein homeostasis; however, chronic ER stress leads to apoptosis. During ER stress, the UPR activates rescue pathways that accelerate protein folding and upregulate molecular chaperones and enzymes to facilitate the degradation of misfolded proteins (Gorman et al., 2012; Hetz, 2012). The three branches of UPR signaling pathways (i.e., IRE1α, PERK, and ATF6) directly or indirectly influence transcriptional and translational responses to the stressor, which either alleviate the source of stress or activate cell death (Ashley Frakes and Dillin, 2017). However, when the UPR declines with age, it is often a consequence of oxidative damage sustained by UPR-regulated chaperone proteins responsible for facilitating protein folding necessary to achieve proteostasis (Rabek et al., 2003; Nuss et al., 2008). A link between protein aggregation and aging is demonstrated by inhibiting protein aggregation and extending lifespan in *C. elegans* suggesting an association between proteostasis and longevity (Silvestre Alavez et al., 2011). The chaperones that appear to be central players in proteome maintenance include BiP, calnexin, and protein disulfide isomerase (PDI), which are observed to decrease both centrally and peripherally with age (Gavilán et al., 2006; Nirinjini Naidoo et al., 2008; Nuss et al., 2008). UPR during senescence (Pluquet et al., 2015) occurs in a wide range of cell types activated by oncogenic Ras (Denoyelle et al., 2006; Dörr et al., 2013; Zhu et al., 2014), xenobiotics (Dörr et al., 2013; Williams et al., 2013; Matos et al., 2015), and DNA damage (Panganiban et al., 2013). It is unclear, however, whether the UPR is a cause or an effect of senescence (Pluquet et al., 2015). The role of the UPR in senescence, therefore, remains controversial, and the mechanisms linking ER and senescence are ripe for interrogation.

## Mitochondria-endoplasmic reticulum contacts (MERCs)

MERCs, the sites of interactions between mitochondria and the ER, contain hundreds of proteins including enzymes, ion channels, and tethering and transport binding proteins (Barazzuol et al., 2021). Membrane fractionation and characterization of contact zones have identified components of the ER that co-purify with mitochondria (Wu et al., 2018) and comprise the biochemical fraction of MERCs known as mitochondria associated membranes (MAMs). MAMs are the sites of interaction between organelles (Vance et al., 1997; Patergnani et al., 2011) and play a role not only in metabolite trafficking and signal transduction but in membrane fusion, mitophagy, and lipid metabolism (Janikiewicz et al., 2018). MAM proteins are broadly classified as protein tethers, ion channels, or enzymes (Wang et al., 2019) and are vulnerable to age-related dysregulation that occurs as a consequence of loss or disruption of critical metabolic fluxes (Janikiewicz et al., 2018; Moltedo et al., 2019; Ziegler et al., 2021). It remains unclear whether MAM composition is implicated in the aging process (Janikiewicz et al., 2018).

Although mechanisms of MERC-mediated senescence have not been fully elucidated, MERCs are implicated in dysregulation of Ca^2+^ exchange and flux (Giacomello and Pellegrini, 2016), which may potentiate cellular senescence by increasing mitochondrial Ca^2+^ concentration. The loss of protein tethers that control Ca^2+^ and phospholipid fluxes disrupts mitochondrial respiration and likely contributes to the aging process (Patergnani et al., 2011). Among the MAMs across MERC sites that regulate Ca^2+^ flux, the tethering mitofusin proteins MFN1 and MFN2 act as conduits from the mitochondria to the ER, where flux of Ca^2+^ into the mitochondria is controlled by the IP3R-DJ-1-GRP75-VDAC complex (Kaul et al., 2003; Son et al., 2017; Liu et al., 2019).

### Ca^2+^ reservoirs, gradients, and flux in the MERC space

Ca^2+^ is critical for mitochondrial function and energetics, and the ER acts as a reservoir for Ca^2+^ by maintaining a high Ca^2+^ gradient with the aid of the ATPase SERCA (Wang et al., 2019). Ca^2+^ is not only a cofactor for enzymes in cellular respiration but is also trafficked across MERCs (Szibor et al., 2020). The MERC space accommodates Ca^2+^ signal transfer between the ER and mitochondria *via* IP3 receptors (IP_3_R) permitting overall cell survival (Bartok et al., 2019). Proteins in the MERC space (e.g., IP_3_R and VDAC1) control flux of Ca^2+^ into mitochondria (Sander et al., 2021). In addition to Ca^2+^ trafficking, MERCs modulate: 1) bidirectional lipid transfer that is critical to steroid synthesis and membrane maintenance; 2) mitochondrial quality control, which employs pro-fusion proteins (e.g., MFN1 and 2, and OPA1) to drive mitophagy (e.g., PINK1 and parkin); and 3) the ER stress response and the UPR, involving PERK, among other stress-responsive proteins (Janikiewicz et al., 2018; Veeresh et al., 2019; Picca et al., 2020; Wilson and Metzakopian, 2021).

Ca^2+^ serves an especially important role as a first and second messenger in signal transduction and as a cofactor of enzymes in mitochondrial cellular respiration (Fink et al., 2017). Dysregulation of inter-organellar Ca^2+^ homeostasis impacts metabolic fluxes, results in apoptosis, and is likely a mechanism of action in diseases arising from ER stress. Proteins that are important in Ca^2+^ shuffling include MFN2, which is found on the outer membrane of the cell and aids in the transport of Ca^2+^ from the mitochondria to the ER. MFN2 is a negative regulator of MERCs; therefore, ablation of MFN2 leads to increased Ca^2+^ shuttling from the ER to mitochondria (Leal et al., 2016). MFN2, which is enriched in MAM fractions, regulates ER morphology by tethering the ER and mitochondria. The movement of Ca^2+^ from a storage organelle to an energy-producing organelle is critical to cellular metabolic homeostasis, and interrogation of this process with MSI will likely reveal mechanisms of aging and age-related diseases associated with dysregulated Ca^2+^ fluxes. Understanding the molecular mechanisms of diseases arising from ER stress may re-contextualize the pathophysiology of diseases of aging, including cancers (Lin et al., 2019), Parkinson’s disease (Surmeier et al., 2017), type 2 diabetes (Burgos-Morón et al., 2019), Alzheimer’s disease (Uddin et al., 2021), and muscle disorders (Gommans et al., 2002), among others.

### Pyridine nucleotide fluxes

NAD(P)^+^/NAD(P)H and the pyridine nucleotide derivatives of NAD(P)^+^ (e.g., NADP and ADPR) potentiate Ca^2+^ efflux from the ER (Churchill and Galione, 2001; Mándi et al., 2006) and also serve as redox cofactors in enzymatic reactions that produce ATP and synthesize fatty acids, phospholipids, select amino acids, and steroids (Wang et al., 2019). NADPH reduces oxidized precursors during the synthesis of lipids, deoxyribonucleotides, and proline, and NAD^+^ removes electrons during the metabolism of sugars and fats, producing the NADH required to make ATP. Although the ratio of NADPH/NADP^+^ is high in all cell types to accommodate biosynthesis and the oxidative stress response, the NADH/NAD^+^ ratio depends on nutrient availability, differs across organelles, and depends upon localization of metabolic enzymes in cellular compartments (Hosios and Vander Heiden, 2018). Vesicles and membrane contact sites are responsible for the trafficking of metabolites, lipids, and proteins, thereby facilitating adaptation of cells to changing extracellular conditions (Cohen et al., 2018). Cellular homeostasis, however, requires sufficient levels of molecules such as NAD(P)^+^/NAD(P)H, which declines with age (McReynolds et al., 2020). How organelles affect the NAD^+^ levels in a cell and how aging alters this process is an area that remains open to interrogation and MSI may be useful for greater resolution of more nuanced roles of NAD^+^.

## Visualizing cellular and subcellular metabolism in age-related disease models

Current methods for the visualization of interorganelle spaces like MERCs include electron microscopy (EM), which provides a high-resolution image of cellular or tissue ultrastructure that is ideal for study of organelles, viruses, and macromolecules. An array of EM approaches offers detailed resolution of cellular architecture. Scanning EM (SEM), for example, is a high-resolution imaging technique using a focused beam of electrons to resolve surface topography. Transmission electron microscopy (TEM) is commonly used for high-resolution 2D micrographs (Garza-Lopez et al., 2021; Lam et al., 2021); however, there are several 3D imaging approaches which permit visualization of organelles including serial block-face scanning electron microscopy (SBF-SEM), focused ion beam scanning electron microscopy (FIB-SEM), and cryo-EM among others. The main difference between SBF-SEM and FIB-SEM is that the former uses a beam of elections and latter uses a beam of gallium ions, permitting the resolution of mitochondria cristae structure or organelle-organelle interactions such as MERCs. Electron tomography, an extension of TEM that includes cryogenic electron microscopy (Cryo-EM) improves visualization of organelles by avoiding crystallization (Xu Benjin and Ling, 2020). Other techniques, including optical and fluorescence microscopy, provide visualization of the contact sites and Ca^2+^ signaling in MERCs using luminescent or fluorescent Ca^2+^ sensors. Compared to these other methods, MSI is unique in capturing individual cells, cellular interactions, and the chemical microenvironment without requiring the dissociation of tissues.

Previous techniques using 2D static images to visualize MERCs have been described (Giacomello and Pellegrini, 2016). Whereas 2D static images provide lower quality images of MERC interactions, SBF-SEM provides 3D visualization of MERC interactions but not live images of ER and mitochondrial dynamics. Similarly, whereas immunogold labeling provides a snapshot of the location of a protein in a specific area of the electron micrograph, live light microscopy in contrast shows real-time changes in cristae and MERC dynamics at high resolution. Live light microscopy, however, requires fluorophores that often produce photobleaching and poor image capture. The combination of electron and light microscopy—also called correlative light and electron microcopy (CLEM)—is powerful but is limited by the number of fluorophores and a requirement of imaging at low temperatures. The CLEM workflow for 3D reconstruction of images provides insight into spatial relationships and specific proteins. However, CLEM permits imaging of only a few proteins at one time. Imaging technologies that can capture metabolic flux across organelles are still lacking.

The tissue microenvironment comprises heterogeneous populations of cells through which metabolites flow, interact, and shape the cellular biochemical milieu, and tissue function depends on nutrient supply and a sustained flux of metabolites. Mapping the intra- and inter-cellular metabolic flux may be an approach for elucidating the aging process in parenchymal cells as well as determining how the chemical environment shapes the tissue microenvironment. Such mapping will require the application of single-cell omics to sample the spatial landscape of cells and organelles to accurately describe metabolic heterogeneity. Currently there is no *in vivo* method to visualize protein or metabolite trafficking in real time. However, MSI images addresses this deficit by simultaneously imaging multiple proteins and metabolites and measuring the distribution of proteins by quantifying peptide markers of the protein (Hale and Cooper, 2021). Although the complex data generated from MSI analysis of protein complexes typically requires computational information processing, use of multiple labeled isotopes at once offers improvements over traditional methods by imaging the spatial distribution of the cell’s chemical microenvironment. Taken together, whereas EM-based technologies provide insight into the cellular landscape, structures, and connections between various organelles, MSI-based technologies permit visualization of biomolecules in the sampled pixel or voxel within the cell.

MSI-based single-cell omics have been used to elucidate the biochemical variations among individual cells in the tissue microenvironment or in cell culture in order to better understand the cellular landscape of immunometabolism (Jackson et al., 2020; Manzo et al., 2020; Rovira-Clave et al., 2021) and cellular dysregulation in cancer (Zhang and Vertes, 2018; Tian et al., 2021b) and microbial resistance (Tian et al., 2017b). However, there are few applications of MSI in research on aging. Starr et al. (2016) report the use of SIMS (secondary ion mass spectrometry) to assess physiological changes in the stratum corneum of photo-aged human skin. In addition to an altered spatial distribution of sterol cholesterol sulfate, there is more lignoceric acid (C24:0) and hexacosanoic acid (C26:0) in photo-aged human skin, which have not been linked previously to aging skin. These findings provide insights not only into novel aging mechanisms, but also could inform the design of both pharmaceutical and topical cosmetic products to counter photoaging. Additionally, SIMS imaging coupled with stable isotope labeling was used to trace DNA synthesis and *de novo* lipogenesis to measure cell proliferation and lipid turnover. Imaging data show an age-dependent decline in new adipocyte generation and adipocyte lipid turnover likely mediated by an age-related decline in insulin-like growth factor-1 (IGF-1) (Guillermier et al., 2017).

Although studies of aging using MSI demonstrate chemical variations across individual cells, the integration of different omics in single cells for a comprehensive multilevel understanding of the molecular mechanism is challenging. Recently, improved single cell resolution has been achieved by improved MSI methods and workflows. For example, with MALDI, improved laser focus, matrix application, and molecular ionization have made the combination of metabolomics and proteomics possible in single cells (Rubakhin et al., 2003; Zavalin et al., 2015; Kompauer et al., 2017; Wang et al., 2022). Thus, MSI is an analytical tool that can image multiple untagged biomolecules to monitor metabolic pathways originating from cellular/subcellular compartments. This technology not only improves upon existing approaches but leverages substantial spatial resolution to interrogate challenging questions in the field of aging, including: 1) the intraorganellar locale of suppressed *de novo* lipogenesis observed during ER stress in aging tissues (Ward et al., 2022); 2) mitochondrial ATP dynamics in senescent cells (Depaoli et al., 2018); 3) metabolic fluxes through multiple single-cell types in tumor tissue (Jeong et al., 2017); and 4) the subcellular localization of lipid storage in various metabolic diseases (Kim et al., 2016). The value of MSI rests in the layers of biochemical data that can be mined with precision from individual cells in tissue without the need for tissue dissociation. MSI offers a comprehensive view of aging on the cellular and subcellular level with the potential to generate new hypotheses and identify novel therapeutic targets. The failure to achieve sufficient molecular coverage, spatial resolution, and non-cryogenic analysis has hindered the application of spatial lipidomics/metabolomics at subcellular resolution. Other challenges that all MSI tools face include metabolic isomer separation, quantification within complex biomatrices, protein imaging, low-concentration biomolecules. The development in the technology and methodology have been adapted to solve the issues, such as integration of ion mobility for isomer imaging (McLean et al., 2007; Zandkarimi et al., 2019), external standards for quantification calibration (Landgraf et al., 2011), novel desorption sources (e.g., LESA and GCIB) for visualization of protein and trace molecules (Sarsby et al., 2015; Sheraz et al., 2019; Tian et al., 2021a). MSI can also be used to understand general disease states in MERCs and how the complex metabolomic and protein makeup of MERCs is altered during pathogenesis.

In the following sections, we will consider the role of the mitochondria, the ER, MERCs, and the metabolic changes that occur in these structures in various diseases associated with aging. We propose that MSI can provide lipidomics/metabolomics information at a subcellular level to better understand the role that metabolic changes in the mitochondria, the ER, and MERCs play in the development of these diseases.

## Exploring diseases of aging at the organellar level: Outstanding questions and opportunities

### Neurodegenerative disorders

Diseases of aging occur from oxidative stress, mitochondrial mutations, misfolded proteins, or failure of defective organelles to turn over (Moltedo et al., 2019); therefore, the ER and ER contact sites are potentially important targets of aging. ER stress, leading to the disruption of MERCs, is observed in models of Alzheimer’s disease and Parkinson’s disease (Remondelli and Renna, 2017) and is a hallmark of other neurodegenerative disorders, including Huntington’s disease and amyotrophic lateral sclerosis (ALS). Production of the β-amyloid peptide in Alzheimer’s disease, which is processed to the amyloid precursor protein by γ-secretase, increases the physical interactions of MERC sites and augments Ca^2+^ shuttling between the ER and mitochondria (Zampese et al., 2011). Likewise, mutant forms of α-synuclein and parkin that are associated with the pathophysiology of Parkinson’s disease compromise Ca^2+^ transport across MERCs and lead to increased autophagy and diminished mitochondrial dynamics, respectively (Calì et al., 2012; Calì et al., 2013). ROS and apoptotic signals are regulated by the MERC tether PERK (Verfaillie et al., 2012), which when upregulated in Alzheimer’s disease and Parkinson’s disease likely results in increased cell death and is correlated clinically with memory loss (Ma et al., 2013). Interestingly, inhibition of PERK reduces MFN-2 contact with the ER and alleviates ER stress (Muñoz et al., 2013; Celardo et al., 2016). Visualizing the locale of proteins associated with ER stress may provide insight into mechanisms of neurodegeneration and inform therapeutic strategies for the treatment of neurodegenerative disorders.

Maintaining Ca^2+^ homeostasis is also a strategy to alleviate ER stress in neurons. Despite limited *in vivo* studies in models of neurodegenerative diseases, *Drosophila* studies show that targeting pro-fusion MERC proteins critical to Ca^2+^ homeostasis in mitochondria (e.g., PINK1) promotes the survival of neurons (Lee et al., 2018). Recent studies in dopamine neurons, which are targeted in Parkinson’s disease, suggest that ER increases its levels of NADH and NADPH in response to metabolic stress in mitochondria. This apparent crosstalk between stress-responsive organelles suggests a coordinated response involving the sensing and shuttling of redox molecules across MERCs (Tucker et al., 2016). An example of a redox-sensitive response shared across MERCs is observed in MERCs of fibroblasts derived from individuals with ALS with a complex I deficiency. In these cells, increased NADH/NAD^+^ ratios in mitochondria appear to activate the UPR in the ER, suggesting that metabolic remodeling of redox-sensitive molecules triggers a stress response across organelle contact sites (Straub et al., 2021). Motor neurons affected by ALS, unlike fibroblasts, may be less responsive to an adaptive stress response, given the differences in nutrient uptake between fibroblasts and neurons. Metabolic remodeling might be captured in real time through spatial resolution by MSI and provide further elucidation of the ER stress response across different cell types.

Astrocytes with a high metabolism react to the stress of inflammation or neuronal injury by upregulating Ca^2+^ signaling, which can be a predictor of disease severity (Shigetomi et al., 2019). Ca^2+^ is mobilized by the activation of the ryanodine receptor in the ER by cADPR produced by CD38-mediated breakdown of NAD^+^ (Yamasaki et al., 2005). Increased Ca^2+^ flux results in greater ATP production and mitochondrial antioxidant potential (Park et al., 2021). The role of NAD(H) and NADP(H) in MERCs is not well understood; therefore, determining the location and trafficking of these molecules across MERCs using a spatial omics approach may clarify the role of redox sensitive pyridine nucleotides in homeostasis and disease. The voltage-dependent anion channel (VDAC) interaction with IP_3_R and deglycase (DJ-1), which is regulated by glucose-related protein 75 (GRP-75) (Basso et al., 2020), is critical to Ca^2+^ homeostasis in MERCs. While the importance of the tethering of MERCs for Ca^2+^ homeostasis is known, the presence of DJ-1 has only recently been discovered (Liu et al., 2019; Basso et al., 2020). MSI could provide an estimation of the relative abundance of specific proteins in diseases.

### Chronic metabolic disorders

Metabolic syndrome is a constellation of cardiometabolic risk factors that include obesity, insulin resistance, dyslipidemia, and hypertension (Moltedo et al., 2019). During metabolic homeostasis, the ER and mitochondria serve as nutrient sensors that modify the distance of MERC sites to adapt to changes in nutrient type and availability, also known as metabolic flexibility (Sood et al., 2014). Loss of this adaptive capacity compromises metabolic homeostasis leading to chronic metabolic diseases (Galgani et al., 2008). Dysregulation of MERCs leads to inter-organellar miscommunication resulting in mitochondrial Ca^2+^ overload, impaired insulin signaling by ER stress, and lipid dysregulation and may play a yet determined key role in metabolic homeostasis (Morgado-Cáceres et al., 2022). Investigating the molecular mechanisms of metabolic flexibility using a combination of imaging and omics through MSI may improve upon approaches that have been taken historically to measure physiological adaptability (Bret Goodpaster and Sparks, 2017).

Insulin resistance (IR) is perhaps the best-characterized example of miscommunication between the ER and mitochondria (Arruda et al., 2014; Rieusset et al., 2015; Theurey et al., 2016; Rieusset, 2018; Dia et al., 2022). IR or impaired insulin sensitivity results in decreased glucose uptake by cells in type II diabetes and obesity and can be attributed to mitochondrial dysfunction leading to the release of Ca^2+^ from the ER and ER stress (Lim et al., 2009) and dysregulation of MERCs in response to palmitate, high fat, or high sucrose (Tubbs et al., 2014; Tubbs et al., 2018a; Tubbs et al., 2018b; Beaulant et al., 2022) in the diet. Several proteins that reside in the MERCs may be involved in obesity-induced IR including MFN2 (Sebastián et al., 2012; Gan et al., 2013), IP_3_R1 (Arruda et al., 2014), PACS2 (Arruda et al., 2014), PDK4 (Thoudam et al., 2019) as well as many other proteins. It has been proposed by Arruda et al. (2014), that increased MAM formation drives several processes that lead to impaired insulin action and aberrant glucose metabolism. Together these finding suggest that disruption of MERCs and resident MAM proteins, are important for understanding organelle dysfunction and possibility that restoring MERCs proper function may be useful for future therapeutics (Rieusset, 2018).

In addition to regulating insulin, MERCs affect enzymes that regulate lipid metabolism (Cheng et al., 2020). Phospholipid exchange between ER and mitochondria requires intact MERCs and is required to synthesize phosphatidylserine (PS), phosphatidylethanolamine (PE), and phosphatidylcholine (PC) (Szymański et al., 2017). An increase in the ratio of PC:PE inhibits ER Ca^2+^ transport and induces ER stress in a murine obesity model (Fu et al., 2011). The localization of PS synthesis at contact sites and the shuttling of PS from the ER to the mitochondria by MFN2, which is the rate-limiting step in PE synthesis, provides the optimum PC:PE ratio for cell function. An altered ratio disrupts cell membrane integrity and potentiates steatohepatitis (Li et al., 2006). In mice, the accumulation of fat in the liver decreases NAD^+^, triggers mitochondrial dysfunction, and curtails ATP production (Gariani et al., 2016). Supplementation with the NAD^+^ precursor nicotinamide riboside (NR) reverses fatty liver disease in mice through SIRT1-and SIRT3-dependent UPR in mitochondria, suggesting that boosting NAD^+^ could reverse mitochondrial dysfunction (Gariani et al., 2016). The ability to simultaneously visualize MERC distance and measure flux of phospholipids from ER to mitochondria using MSI may enhance understanding of IR at an organellar level.

### Muscle disorders

Skeletal muscle responds to a continuum of stressors that challenge metabolic homeostasis and either forge an adaptive change or result in pathogenesis. Evidence is mounting in support of a role for contact sites between mitochondria and the sarcoplasmic reticulum (SR), a specialized form of ER charged with Ca^2+^ storage, Ca^2+^ mobilization, and signaling. Disrupted transport of Ca^2+^ across MERCs has emerged as an important feature of muscle disorders. As a contractile tissue, skeletal muscle relies upon availability of ATP, which requires local transport of Ca^2+^ between SR and the mitochondria contacts. Ca^2+^ is required in oxidative metabolism and acts as a cofactor of TCA enzymes (e.g., isocitrate dehydrogenase, a-ketoglutarate dehydrogenase, pyruvate dehydrogenase) and synthesis of reducing equivalents (e.g., NADH, FADH2). Whereas IP3R (IP3R1, IP3R2, IP3R3) modulates release of Ca^2+^ stored in the ER/SR, VDACs mediates the flux of Ca^2+^ into mitochondria (Mikoshiba, 2015; Bartok et al., 2019; Fan et al., 2022; Schmitz et al., 2022). An additional MAM-enriched protein called SEPN1 has been identified at the ER/SR interface (Filipe et al., 2021). SEPN1 appears to enhance ER/mitochondria contacts, calcium flux, and oxidative phosphorylation and its dysregulation has been implicated in severe muscle weakness. The movement of calcium from a storage organelle to an energy producing organelle is critical to cellular metabolic homeostasis and interrogation of this process is likely to shed greater light on mechanisms of muscle disorders.

While Ca^2+^ flux into the mitochondria is essential for normal metabolism, an overload of Ca^2+^ activates the mitochondrial permeability transition pore, resulting in mitochondrial swelling and the dissemination of apoptotic signals (Zhou et al., 2022). To counter the problem of Ca^2+^ toxicity leading to cell death, the tethering protein MFN2, which is localized to the outer mitochondrial membrane and ER surface, protects mitochondria by creating space within MERCs (Filadi et al., 2015). de Brito and Scorrano (2008) demonstrated MFN2 to be a bonafide tether in MERCs. Conversely, this discovery was challenged (Filadi et al., 2015) by Filadi et al.; however, Naon et al. (2016), reconfirmed MFN2 as a MERC tether by providing evidence that Mfn2 ablation increases MERC distance (Sebastián et al., 2012). Additionally, Naon et al. (2016) showed that MFN2 ablation causes reduction of mitochondrial Ca^2+^ uptake but doesn’t affect mitochondrial Ca^2+^ uniporter complex, further confirming MFN2 as a critical tether important for MERC distance. Moreover, MFN2 has a critical role in glucose homeostasis and insulin signaling in skeletal muscle (Sebastián et al., 2012). This suggests that MFN2 in skeletal muscle is an important regulator of mitochondrial dynamics and MERCs. MFN2 dimerizes with itself on the outer mitochondrial membrane but also interacts with MFN1 on the inner mitochondria membrane allowing for interaction with inner membrane fusion proteins such as OPA1. OPA1 functionally requires MFN1 further confirming the distinct roles between the two proteins (Sood et al., 2014). Interestingly, ablation of OPA1 induces ER stress, which in turn upregulates MFN2, as a key MERC tethering protein (Pereira et al., 2017). This suggests that as a possible compensatory mechanism for MFN2 upregulation during loss of OPA1 in skeletal muscle. MERC ultrastructure may also contribute to the pathophysiology of certain muscle disorders. For example, MERCs decrease after endurance exercise (Merle et al., 2019) but increase in aging (Marzetti et al., 2016; Sebastián et al., 2016; Picca et al., 2017), suggesting that the ultrastructure of MERCs in muscle may respond differentially to stressors.

Although MERCs play a role in some muscle myopathies, their function is less clear in sarcopenia, a myopathy in the elderly that is accompanied by a progressive increase in body fat (Hsu et al., 2019) and risk of metabolic syndrome (Umegaki, 2015). Several muscle disorders have been linked to a failure of Ca^2+^ regulation by MERCs, including Duchenne muscular dystrophy (DMD) and sarcopenia. DMD results from the defective cytoskeletal protein dystrophin. In dystrophin-deficient muscle, Ca^2+^ homeostasis is dysregulated, which results in aberrant Ca^2+^ signaling and increased ROS production (Cully and Rodney, 2020). Similarly, mutations in ryanodine receptor (RyR1) lead to severe muscle myopathies, decreasing SR Ca^2+^ amplitudes (Lee et al., 2017). Such ryanodine receptors are important for Ca^2+^ leakage and mishandling, playing a major role in diseases such as DMD (Meyer et al., 2021). Ca^2+^ levels in dystrophin-deficient skeletal muscle are regulated by the SR membrane-bound ryanodine receptor, which promotes Ca^2+^ release (Meyer et al., 2021), and the SR membrane-bound Ca^2+^ salvaging proteins SERCA1 and SERCA2a, which in dystrophin deficiency fail to scavenge excess Ca^2+^ (Schneider et al., 2013; Voit et al., 2017).

Ca^2+^ dysregulation can lead to oxidative stress and ROS production. To counter ROS, antioxidant enzymes rely on NADPH as a source of reducing equivalents. In muscle SR, hexose-6-phosphate dehydrogenase (H6PD) reduces NADP^+^ to produce the NADPH needed for steroidogenesis, and H6PD-deficient skeletal muscle exhibits a myopathy characterized by abnormal SR structure (Lavery et al., 2006; Rogoff et al., 2010), activation of stress-induced UPR (Bujalska IJ et al., 2008), and dysregulation of Ca^2+^ metabolism (Ying, 2006). Therefore, H6PD is important not only for steroidogenesis but also for normal ER/SR redox balance. H6PD knockout mice upregulate NRK2-mediated biosynthesis of NAD^+^ from NR in response to dysregulated NADPH homeostasis in muscle SR and impaired metabolism in mitochondria (Doig et al., 2020). Curiously, NR supplementation of H6PD knockout mice fails to reverse the muscle myopathy phenotype, despite improvement following NR supplementation in a mouse model of mitochondrial myopathy (Khan et al., 2014). The role of H6PD modulation of NADPH in whole-cell redox and metabolism remains to be elucidated and may be visualized and quantified by MSI.

The changes in insulin signaling, loss of proteostasis, and inflammation characteristic of sarcopenia are likely mediated by mitochondrial dysfunction (Coen PM et al., 2019; Ferri et al., 2020). Mitochondrial dysfunction leads to: 1) loss of mitochondrial quality control (Joseph et al., 2013); 2) increased activity of the ryanodine Ca^2+^ channel receptor, resulting in Ca^2+^ overload and mitochondria permeability transition pore activation (Andersson et al., 2011); 3) Ca^2+^-induced decreased proteolysis and the formation of toxic proteins (Baehr et al., 2016); and 4) ROS leakage due to mitochondrial dysfunction (Shally and McDonagh, 2020). These findings support a role for MERCs in the pathogenesis of sarcopenia (Sebastián et al., 2016; Wiedmer et al., 2021). However, we have a very limited understanding of sarcopenia development, due to lack of appropriate animal models and the difficulty in separating “pure sarcopenia” from other age-related comorbidities (Christian CJ and Benian, 2020).

### Atherosclerosis

The pathogenesis of the age-related development of atherosclerosis (Finney et al., 2017), an inflammatory disease of the arteries leading to fibrosis, clotting, and increased cardiovascular risk, can be tied to dysfunction of mitochondria-associated ER membrane formation in smooth muscle (Bennett et al., 2016; Perrotta, 2020; Wang et al., 2021) and endothelial cells (Nguyen et al., 2012). One mechanism of MERC-mediated atherosclerosis depends on the tethering protein PACS2 to transfer Ca^2+^ from the ER to mitochondria, resulting in Ca^2+^ overload, loss of mitochondrial membrane potential, increased production of ROS, the release of cytochrome c, and, eventually, apoptosis (Moulis et al., 2019; Yu et al., 2019). Oxidized low-density lipoprotein also induces inflammation in endothelial cells, thereby recruiting the NLRP3 inflammasome to contact sites with the activation of vascular remodeling (Zeisbrich et al., 2018). The NLRP3 inflammasome is also activated by ATP, ROS, ER stress, Ca^2+^ signaling, and mitochondrial dysfunction (Strowig et al., 2012; Chen and Chen, 2018). Ca^2+^ mobilization *via* the cell-surface type II transmembrane glycoprotein CD38 is critical for the activation of the NLRP3 inflammasome (Li et al., 2020). CD38 is a NADase that metabolizes nicotinamide nucleotides (NAD^+^ and NMN) to ADPR and cADPR (Hogan et al., 2019). cADPR mobilizes Ca^2+^ by activating the ryanodine receptor in SR (Venturi et al., 2012). Recent data in a diabetic mouse model demonstrate that CD38-mediated Ca^2+^ signaling leads to mitochondrial damage, the release of mitochondrial DNA, and activation of NLRP3 inflammasome in vascular smooth muscle (Li et al., 2020). The tissue specific locale of CD38-mediated ADPR and cADPR production and subsequent Ca^2+^ mobilization are likely candidates for spatially resolved omics available through MSI. To this end, the NAD^+^ consumer CD38 has potential as a therapeutic target in atherosclerosis.

## Perspectives

MSI is an analytical tool that has the capability to image multiple untagged biomolecules for the purpose of monitoring metabolic processes originating from cellular/subcellular compartments. This technology not only improves upon existing approaches, but leverages its substantial spatial resolution to interrogate challenging questions in the field of aging including: the intraorganellar locale of suppressed *de novo* lipogenesis observed during ER stress in aging tissues (Ward et al., 2022); mitochondrial ATP dynamics in senescent cells (Depaoli et al., 2018); metabolic fluxes through multiple single cell types found in tumor tissue (Jeong et al., 2017); and the subcellular localization of lipid storage in various metabolic diseases (Kim et al., 2016). The merit of MSI lies in the layered biochemical data that can be mined with precision from individual cells in tissue without the need for tissue dissociation. MSI offers a comprehensive view of aging on the cellular and sub-cellular level with the potential to generate new hypotheses and identify novel therapeutic targets.
